# User Perspectives of Mood-Monitoring Apps Available to Young People: Qualitative Content Analysis

**DOI:** 10.2196/18140

**Published:** 2020-10-10

**Authors:** Emily Widnall, Claire Ellen Grant, Tao Wang, Lauren Cross, Sumithra Velupillai, Angus Roberts, Robert Stewart, Emily Simonoff, Johnny Downs

**Affiliations:** 1 Institute of Psychiatry, Psychology and Neuroscience Kings College London London United Kingdom

**Keywords:** mood monitoring, engagement, mobile applications, mHealth, mental health, smartphone, qualitative research, mobile phone

## Abstract

**Background:**

Mobile health apps are increasingly available and used in a clinical context to monitor young people’s mood and mental health. Despite the benefits of accessibility and cost-effectiveness, consumer engagement remains a hurdle for uptake and continued use. Hundreds of mood-monitoring apps are publicly available to young people on app stores; however, few studies have examined consumer perspectives. App store reviews held on Google and Apple platforms provide a large, rich source of naturally generated, publicly available user reviews. Although commercial developers use these data to modify and improve their apps, to date, there has been very little in-depth evaluation of app store user reviews within scientific research, and our current understanding of what makes apps engaging and valuable to young people is limited.

**Objective:**

This study aims to gain a better understanding of what app users consider useful to encourage frequent and prolonged use of mood-monitoring apps appropriate for young people.

**Methods:**

A systematic approach was applied to the selection of apps and reviews. We identified mood-monitoring apps (n=53) by a combination of automated application programming interface (API) methods. We only included apps appropriate for young people based on app store age categories (apps available to those younger than 18 years). We subsequently downloaded all available user reviews via API data scraping methods and selected a representative subsample of reviews (n=1803) for manual qualitative content analysis.

**Results:**

The qualitative content analysis revealed 8 main themes: accessibility (34%), flexibility (21%), recording and representation of mood (18%), user requests (17%), reflecting on mood (16%), technical features (16%), design (13%), and health promotion (11%). A total of 6 minor themes were also identified: notification and reminders; recommendation; privacy, security, and transparency; developer; adverts; and social/community.

**Conclusions:**

Users value mood-monitoring apps that can be personalized to their needs, have a simple and intuitive design, and allow accurate representation and review of complex and fluctuating moods. App store reviews are a valuable repository of user engagement feedback and provide a wealth of information about what users value in an app and what user needs are not being met. Users perceive mood-monitoring apps positively, but over 20% of reviews identified the need for improvement.

## Introduction

Young people are leaders in adopting new technology, with recent statistics highlighting that 96% of those aged 16-24 years own a smartphone [1], and mobile phone usage among teenagers is increasing more than any other age group [2]. The smartphone revolution has not only changed the way young people communicate and acquire new information [3] but also encouraged a rapid increase of mobile apps with varying functions. This growth is notable in the field of mobile health (mHealth) apps that deliver health and wellness technologies; as of 2017, consumer app stores had >325,000 mHealth apps available for download [4].

There has been increasing interest in using digital technology to administer interventions and monitor mental health symptoms in young people [5]. A particular area of mHealth growth lies in mood-monitoring apps. Mood monitoring is a widely used technique within nonclinical populations, provides insight into the development and trajectory of common mental health difficulties [6-8], and is an embedded technique in existing self-management techniques and evidence-based mental health treatments [9]. Self-tracking mood encourages users to actively engage in their health care management, provides a sense of autonomy [10,11], and increases awareness and self-regulation of emotional well-being [12,13]. There are several reviews exploring mood monitoring in adult populations [14-17]; however, much less is known about their use in child and adolescent populations. Although mood-monitoring apps are potentially cost-effective, accessible, and convenient, there remains a lack of evidence on how acceptable existing mood-monitoring apps are and particularly what features and functions engage younger populations.

This lack of understanding is further compounded by a limited consensus on how to measure user engagement. It is widely acknowledged within the literature that app engagement metrics and reporting remain unstandardized and heterogeneous [18,19]. The term *acceptability* often ranges from proxy markers, that is, adherence rates and utilization data [20], to participants’ experience of burden [16], rather than understanding the features and functions that motivate and satisfy users. A recent systematic review evaluating mobile mood-monitoring apps in young people further demonstrates these inconsistencies [21]. A total of 9 studies of the 25 reviewed considered participants’ perception of the apps, with only 3 studies specifically referring to *acceptability*, which was not explicitly defined; these used utilization and completion data as a proxy, which were interpreted by the authors as demonstrating broad acceptability [16,20,22]. The review demonstrated that young people generally positively perceive mood-monitoring apps and view them as user friendly, convenient, noninvasive, and useful; however, technological difficulties were reported to negatively affect user experience [16,23-25]. The review concluded that very few high-quality studies were available for inclusion and there is a need for more qualitative research to broaden our understanding of factors pertinent to the uptake of mood-monitoring apps.

The adoption of digital tools can also be evaluated by theories of *technology acceptance*, which argue that a person’s intent to use and actual use of a technology is predicated by the person’s perceptions of the technology’s usefulness and ease of use [26,27]. The technology acceptance model (TAM) [26] has been used more recently to explore mHealth whereby *perceived helpfulness*, *perceived ease of use*, *perceived trust,* and *perceived security* were all found to directly influence user intention to use mHealth services [28]. However, the use of the TAM has been criticized for its weaknesses in explaining users’ behavior and oversimplification of user perceptions to *usefulness* and *ease of use* [29].

It is important to gain a deeper understanding of user engagement with mHealth apps, particularly if young people are going to be using publicly available products unaccompanied and on a large scale. It is also crucial that young people are not set up to fail through poorly designed health apps or engagement with well-designed but ineffective digital treatments. Although small-scale qualitative studies have explored young people’s views of mental health apps [30], more extensive research is needed to understand the nuances of user engagement. Written user reviews on mobile app stores contain a wealth of information about user experience and expectations and are a potentially untapped source of information in research, despite being used by smartphone owners to consider whether to download and engage with a given app [31]. We can, therefore, explore rich user reviews to understand what makes a mood-monitoring app acceptable to end users and what features are most prominent in positively reviewed apps.

The analysis of publicly available app reviews has been successfully used in recent literature to investigate user attitudes toward existing apps and their feature requests [32-34]. However, only a handful of studies have analyzed mHealth app reviews. These investigations have tended to assess app content quality for specific disorders [35], app functionality and user experience of a specific intervention [36], or apps targeting medication adherence [37]. Consumer perspectives of apps for bipolar disorder [35] found mostly positive feedback but also a large number of requests for desired functions. Users valued apps that were helpful, supportive, and easy to use and often integrated them into their health management and clinical care. Interestingly, users often did not consider the evidence base or clinical effectiveness of the app. User experience of cognitive behavioral therapy apps for depression [36] found that users valued the app in supporting their mental well-being and used the app as an adjunct to treatment. Concerns were also highlighted, particularly surrounding the importance of privacy, security, and trust. User experience of medication adherence apps [37] again found that users valued customization, the ability to monitor health information, and the ability for apps to support health care visits. Negative user experiences included technical difficulties, confusing app navigation, and inflexibility in the reminder setup.

In this paper, we perform a qualitative content analysis of user reviews to explore what app users consider useful to encourage frequent and prolonged app usage of mood-monitoring apps appropriate for young people.

## Methods

### Data Collection

A systematic review framework was applied to the search, screening, and assessment of apps. We searched the 2 major commercially available app stores: Google Play and Apple iPhone Operating System (iOS) store, by using the application programming interfaces (APIs) on these platforms [38,39]. We first used manual keyword searches to create a set of seed apps, and then, we used a combination of API methodologies to identify and refine a set of relevant mood-monitoring apps on each platform. We then used API data scraping methods to collect all available user-generated reviews for the relevant apps identified. We used a combination of keyword searches to define a set of seed apps, followed by a snowball sampling technique to collect a series of similar apps. A full description of the API data collection methods can be found in Multimedia Appendix 1 [36,40-44].

Apps that met the following criteria were included: (1) self-reported mood-monitoring was the app’s primary purpose, (2) the app was suitable for young people (aged less than 18 years) as described via the app store age rating, and (3) the app was available in English. Apps designed for health conditions other than mood disorders were excluded.

**Figure 1:**
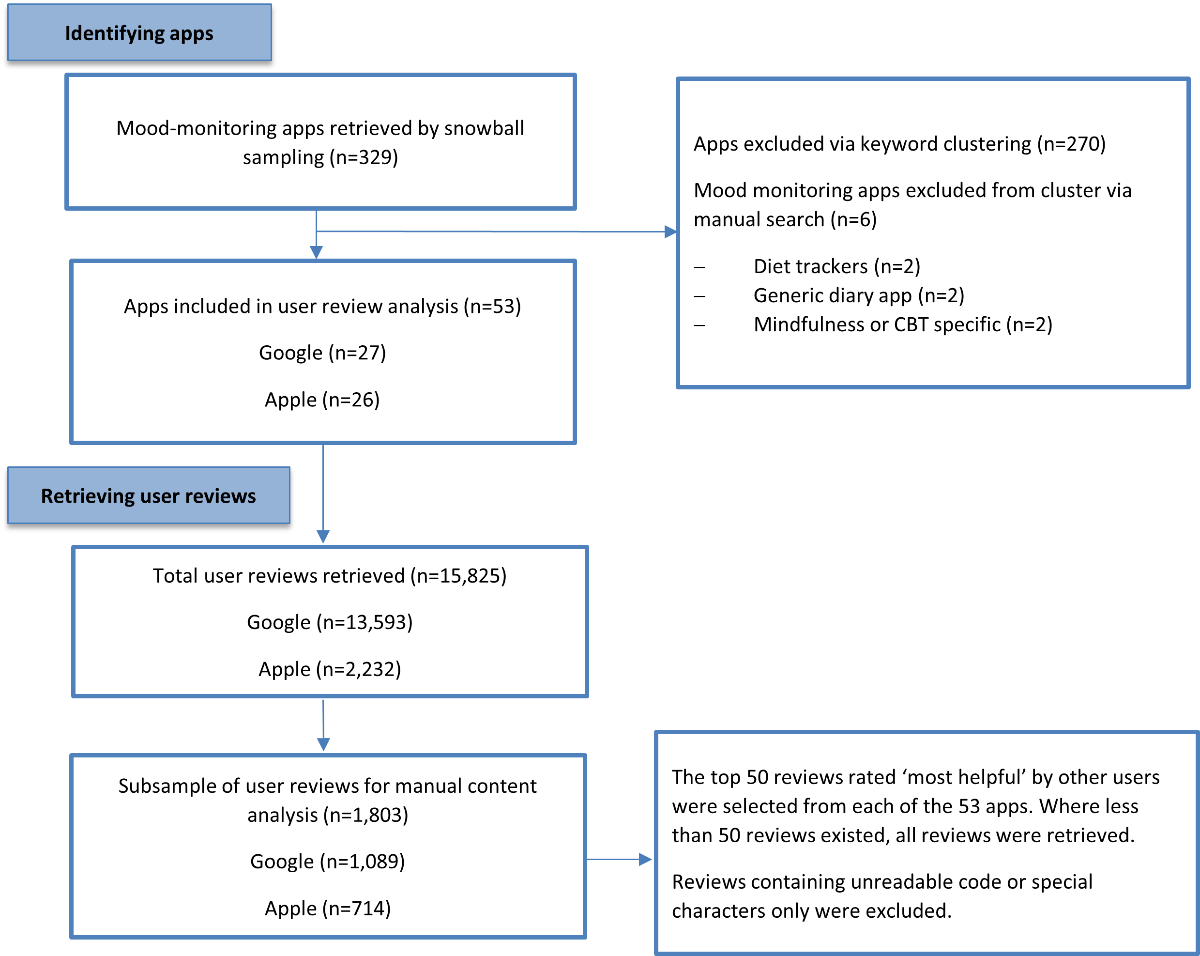
Flow diagram illustrating the selection of apps and user reviews. CBT: cognitive behavioral therapy.

In total, 53 apps had 15,825 reviews. To gain a feasible number of reviews to manually appraise, we systematically selected a subsample of reviews. Both app stores provide different ways of sorting reviews, such as by date (most recent) or by the helpfulness of a review rated by other users (most helpful). After establishing that there was no significant difference between star rating distributions between the two ranking systems (see Multimedia Appendix 1 for full description), we ranked the reviews by *most helpful* and retrieved the top 50 reviews from each app. Where apps had less than 50 reviews, all reviews were collected. This resulted in a subset of 11.39% (1803/15,825) reviews: 1092 Google and 716 iOS. Figure 1 shows a flow diagram detailing the review process and results at each stage.

### Qualitative Content Analysis

Given the exploratory nature of this study, we conducted a qualitative content analysis to interpret themes in the app user reviews. Content analysis techniques have been widely used to understand user-generated review data, particularly in fields of research where existing theory is limited [45-47]. Given the rarity of content analyses of consumer perspectives on mobile apps within the literature, the majority of the analysis used an inductive approach to developing a coding framework [48]. Although the existing literature on analyzing mHealth app user reviews is limited, a smaller-scale deductive approach was carried out by using existing themes drawn from what published research was available to further inform our content analysis framework [11,21,35,49]. Following the guidelines on inductive analysis approaches in previous studies [21,35], we developed a database of coded user reviews. Our approach to the analysis followed established coding techniques [50] across 3 phases: (1) data immersion, (2) data reduction, and (3) interpretation.

A preliminary sample of 500 reviews was randomly selected to ensure adequate coverage of a range of apps. Three coders (EW, CG, and LC) each coded the reviews for positive, negative, or neutral sentiment and noted whether the review highlighted a specific feature of engagement. This allowed the coders to become familiar with the informational content and to generate first-stage concepts.

After initial sentiment and feature identification, the coders developed a preliminary framework to organize codes (code name, description guidelines, and example quotes). Each researcher then used the codebook on an additional 100 randomly selected reviews. All researchers then met to revise, refine, and finalize the codes.

Interpretative notes were made and discussed, particularly around exploring word usage and the range of meanings. A set of themes and subthemes was subsequently revised and reordered during the interpretation phase. A coding framework was finalized, and 2 researchers (EW and CG) then independently coded the subset of user reviews (n=1803).

## Results

### Research Rigor

A substantial level of intercoder reliability across all codes was observed (κ=0.68), with high agreement for the themes adverts (κ=0.85), reminders (κ=0.86), and transparency (κ=0.89). Substantial intercoder agreement was observed for reflecting on mood (κ=0.72), technical (κ=0.72) accessibility (κ=0.71), recommendation (κ=0.79), and recording/representation of mood (κ=0.71). There were moderate levels of intercoder agreement for design (κ=0.61), developer (κ=0.65), health promotion (κ=0.61), flexibility (κ=0.64), social support (κ=0.51), and user requests (κ=0.59). A poor level of intercoder agreement was observed for games/gamification (κ=0.29). As this theme occurred in less than 1% of the reviews, it was subsequently removed from the coding framework.

### Review Sentiment

Of the subset of 1803 user reviews, 1474 (81.7%) had a positive sentiment, that is, featured positive commentary on the app. However, positive reviews often included a contrasting statement, most commonly a user request for an additional feature. A total of 20% of positive reviews were general in nature and did not provide specific details on which features of the app were valued. A total of 8.9% (162/1803) of user reviews had a negative sentiment, and 9.2% (167/1803) of the reviews had a neutral sentiment. Over a third of the reviews with a negative sentiment included user feedback surrounding technical difficulties.

### Description of Apps and Reviews

A total of 14 themes were identified in the data. Eight themes were prevalent in over 10% of the coded reviews (ranging from 34% prevalence to 11%), and 7 themes were present in less than 10% of the reviews (ranging from 7% to 1%). Codes with >10% prevalence were named major themes, whereas codes with <10% prevalence were named minor themes. Multimedia Appendix 2 shows the full coding framework describing all themes, subthemes, and illustrative quotes. Table 1 shows the frequency and percentage presence of all 14 themes within the user reviews. Multimedia Appendix 3 shows all 53 apps included in the analysis as well as app metadata. The included apps spanned 4 different categories: lifestyle (29/53, 55%), health and fitness (20/53, 37%), medical (2/53, 4%), and productivity (2/53, 4%). Across all 53 apps, there was an average star rating of 4.35, with 33 out of 53 apps rated 4.5 stars or above. Out of 53 apps, 47 were free of charge. The 6 apps that charged users ranged from $0.99 to $4.99.

**Table 1:** Prevalence of major and minor themes identified in user reviews (N=1803, categories not exclusive)

Themes^a^		Prevalence of theme in reviews, n (%)
**Major themes**		
	1. Accessibility	614 (34.05)
	2. Flexibility	370 (20.52)
	3. Recording/representation of mood	322 (17.86)
	4. User requests	302 (16.78)
	5. Reflecting on mood	291 (16.14)
	6. Technical feature	284 (15.75)
	7. Design	225 (12.48)
	8. Health promotion	186 (10.32)
**Minor themes**		
	1. Notifications/reminders	130 (7.21)
	2. Recommendation	115 (6.37)
	3. Privacy, security, and transparency	102 (5.66)
	4. Developer	69 (3.82)
	5. Adverts	55 (3.05)
	6. Social/community	38 (2)

^a^In 366 (20.30%) reviews, no engagement feature was coded.

#### Major Theme 1: Accessibility

Over a third of reviews centered around accessibility of the app. Users valued simplicity and frequently praised a simple and straightforward design that was easy to use. Users also frequently praised apps they perceived as fast and efficient; however, they expressed frustration with inefficient or slow apps.

Cost was also an important aspect; overall, users appreciated apps that were free of charge, but users often seemed happy to pay for premium versions if the app met their needs. However, users were frustrated when there were hidden subscription costs or when they had to pay for an app that did not meet their needs. Other users disagreed with developers charging at all for mental health–related apps.

#### Major Theme 2: Flexibility

The second most prevalent theme was flexibility. Users frequently referenced the need for the app to offer personalized and customizable features to suit individual user needs. This theme largely centered on 4 main features, the first of which was the ability for users to create their own personalized emotions or mood descriptions*.* Second was the ability for users to enter as many mood entries as they wished to in 1 day. Third was the ability for users to edit/modify/delete a previous entry, and the fourth one was that users preferred no restrictions/character limits being placed on free text entries.

#### Major Theme 3: Recording/Representation of Mood

How users record and represent their mood within the app was the third major theme. Of particular importance to the user within this theme was the variety of options available to represent mood, linking in with the theme of flexibility. Users often highlighted the complexity of moods and the need for multiple mood entries as well as custom scales*.* Similarly, users often described how a predetermined list of moods or emotions did not allow them to accurately represent their feelings and often requested the ability to elaborate on their mood using free text descriptions in their own words. Interestingly, some users also indicated the need for a balance between choice and specificity, for example, finding it helpful having a list to choose from when feeling confused over their own emotions but also the need to be able to name a mood of their choice*.*

#### Major Theme 4: User Requests

Approximately 1 in 5 reviews contained a request to the app developer. These requests were often a user wish list and requested features to improve their app experience. The most common requests centered on the theme of flexibility and personalization, such as customizable emotions, multiple entries per day, and editing entries.

#### Major Theme 5: Reflecting on Mood

The ability of users to reflect on their mood and mood entries over time was another main feature of engagement. Users particularly valued seeing their mood entries in the form of a graph or diagram. Users described how a visual display of mood over time allowed them to reflect on their good and bad days and value the ability to observe patterns and link moods to particular activities. Users frequently described the positive effect of reviewing their moods and experiences.

#### Major Theme 6: Technical Feature

Technical features largely referred to technical issues within the apps, such as data loss, inability to share mood entries across devices, or difficulties accessing or using the app. The technical features theme, therefore, often referred to barriers to engagement with the app.

Due to the personal nature of data entered into mood-monitoring apps, users had frequent concerns surrounding loss of data and difficulties backing up or saving data. Many users reported experiencing significant amounts of data loss.

The ability to export or share data with different devices as well as with friends, family, and medical professionals was valued by users. When a mood-monitoring app did not include an export or share feature, this was frequently requested by users.

#### Major Theme 7: Design

The design of the app was important to users particularly in terms of the user interface being visually appealing, described by users with terms such as “beautiful,” “pretty,” and “sleek.” Design preferences included a clean, simple design, which was intuitive and easy to navigate. Users valued simplicity and minimal designs over a cluttered screen.

#### Major Theme 8: Health Promotion

Users valued the ability of mood-monitoring apps to facilitate health promotion. Health promotion had 4 categories: (1) the mood-monitoring app itself being therapeutic for users and aiding self-awareness; (2) the ability to share mood entries with health care professionals to aid clinical appointments and facilitate discussions around their mood; (3) the ability for apps to provide psychoeducation, for example, understanding components of cognitive behavioral therapy; and (4) apps including signposting materials to available support services.

#### Minor Theme 1: Notifications/Reminders

Overall, users were positive around the use of notifications and reminders in apps and found this a helpful way of keeping on track with their mood monitoring. Some users even mentioned that a notification would promote a positive thought. It was also important that users were given the option to tailor their notifications/reminders to suit them.

#### Minor Theme 2: Recommendation

Written reviews also consisted of a number of recommendations to other users. These recommendations were indicative of their appreciation and positive experience with mood-monitoring apps. Users would often make recommendations to friends or family members as well as the wider app community. Users would also sometimes mention that their health care professional had recommended the app to them, which was typically followed by a positive review.

#### Minor Theme 3: Security, Privacy, and Transparency

Security and privacy mechanisms within mood-monitoring apps were important to users, and mistrust became an important issue, particularly surrounding the use of Facebook. The lack of openness regarding how and where data were stored was also a concern for some users. Transparency was rarely explicitly mentioned by users (<1% reviews), but it became a significant issue within individual apps, for example*.* Reviewers sometimes implicitly discussed themes of transparency, although this often conflated with trust, security, and privacy. Although transparency does not appear to be a crucial theme for engagement, knowledge of breeches, although rare, is key for rapid disengagement.

#### Minor Theme 4: Developer

App developers were an important factor for users, and comments to developers included praise and thanks, particularly commenting on timely responses from app developers*.* Users also demonstrated frustrations when app developers were not responsive to technical issues within the app, which led to users leaving a negative review surrounding developer communication itself rather than a specific feature of the app.

#### Minor Theme 5: Adverts

The use and frequency of adverts was important to many users who typically preferred apps with no adverts. Users generally disliked intrusive adverts, particularly those that interrupted the design or visual display of the app. Users were happy with being presented with optional adverts, particularly if wanting to support app developers.

#### Minor Theme 6: Social/Community

A number of reviews referred to the *community* aspect. Some mood-monitoring apps provided a peer support network feature, which was generally positively reviewed by users with a sense of listening to others as well as being listened to. Reviews also often included requests from users for app developers to include a support network to be built into apps that did not have one. Where there was a peer network available, some users described feeling limited in the way they were able to offer support. Users wanted to offer encouraging words but felt unable to, for example, some apps would limit user communication to emojis, which some users felt was not encouraging enough.

## Discussion

### Principal Findings

The aim of this study was to summarize and evaluate the main features of engagement within publicly available mood-monitoring apps appropriate for young people (aged less than 18 years) using app store user reviews. To our knowledge, this is the first exploration of consumer perspectives on mood-monitoring apps appropriate for young people using publicly available review data.

User feedback on mood-monitoring apps could generally be summarized by 8 main themes and 6 minor themes. Reviews varied in length, sentiment, and specificity, with many providing detailed and informative feedback about what engages and disengages users in mood-monitoring apps. Although 1 in 5 reviews did not contain a specific feature of engagement, the majority of reviews that did contain a feature of engagement contained multiple themes, demonstrating the complex and multifaceted nature of user needs.

The proportion of reviews containing positive and negative sentiments was similar to previous results in both general and mHealth apps whereby the majority of reviews contained positive sentiment [21,35]. The central positive features of engagement consisted of accessibility and personalization/customization of app content, which are in line with previous findings of user reviews [30,35,36,49]. The main content of negative reviews in this study also supports previous findings, which cite functionality issues, lack of features, and crashing/data loss as the most common complaints [16,21,24,35,36]. These findings are broadly consistent with TAMs, in that users are more likely to adopt apps with high-quality design that is usable (easy to use, simple, and efficient) and useful (ability to reflect on mood and therapeutic features).

Although the majority of reviews had positive sentiments, the number of user requests (1 in 5 reviews) suggests room for improvement in currently available mood-monitoring apps to adequately address user needs. The frequency of user requests is in line with recent findings from user reviews of apps for bipolar disorder [35] and indicates that users have evolving needs and requirements when engaging with health apps. Users also hold an expectation that developers should address their needs and requests, which has also been found in previous literature [35,51,52]. This expectation also supports the findings that app stores serve as a communication channel between users and developers [52]. There appears to be a user-developer community, which highlights the potential for engaging end users throughout the app development process to ensure that the apps meet user needs before being made publicly accessible. User co-design poses obvious advantages for app function, uptake, and use by the target community [53-55].

Themes of accessibility (free and easy use), design (appearance and content), and social support (peers) show similarity with a recent study exploring adolescents’ needs from mental health mobile apps as well as the importance of young people being in control, which is reflected in our theme of recording/representing mood and the significance of users having ownership over how they record their complex and changing moods [51]. Interestingly, social support, however, was much less prominent within this study compared with previous adult and adolescent studies [35,49,56]. This discrepancy could be due to our focus on generic mood-monitoring apps rather than clinical intervention apps or those designed for specific mental health conditions. This finding could also indicate the facilitation of self-management within mood-monitoring apps, which has been demonstrated in previous research, as well as creating a sense of greater control and autonomy around health management [11,49].

Although there may be a sense of self-management and autonomy within personal mood-monitoring apps, several reviews mentioned the benefit of being able to share their mood data with general practitioners, therapists, or counselors. This demonstrates joint partnerships and facilitation of communication between mHealth apps and health care providers. Users mentioned the ease of using app data within their clinical appointments to better communicate their mood over time as well as how different events had been affecting their mood. This two-way communication with health care providers perhaps demonstrates mHealth apps as a complementary tool to facilitate patient-provider relationships, which is in line with previous findings [37,49].

As found in user reviews of bipolar apps [35], there was a clear absence of discussion of scientific quality within user reviews. This also relates to previous qualitative research whereby users were motivated by information about whether or not an app would help them, but this information was not necessarily evidence based [30]. Again, this may be due to the study focusing on generic mood-monitoring apps, but it could also represent a disinterest in the scientific basis of mental health apps or a lack of health app regulation knowledge among users. It could also suggest that users implicitly trust apps that are publicly available on the app store, which highlights the importance of mHealth app literacy among users regarding evidence and data privacy.

The results of this study demonstrate a range of features that engage users in mood-monitoring apps but also highlight existing barriers that may prevent successful engagement. The positive features of engagement found in this paper include personalization and customization, a simple and intuitive design, features allowing users to reflect on their mood, and the facilitation of both self-management and communication with health care providers. The main barriers to engagement include concerns around privacy and security and technical difficulties surrounding data loss and app bugs/errors.

### Limitations

Our results should be considered in light of the following limitations. First, the data used in this study were publicly available reviews. Our sampling frame for contributors and the representativeness of the views expressed are unknown. User reviews on app stores do not provide demographic data; therefore, we are not aware of the age of users submitting reviews. Although we based this study on apps available to those aged less than 18 years, the user reviews analyzed may be from a wide variety of age ranges, including adults. This is a limitation of using publicly available app data; hence, the engagement features we reviewed cannot be generalized to youth populations specifically.

Another limitation of looking at written user reviews is that they lack data surrounding user retention rates and periods of user engagement. It is, therefore, not possible to determine if reviews have been written after limited or extensive use of an app. Further research is needed to explore additional variables such as level of usage, understanding intent to start using mood-monitoring apps, and social influences.

This was an exploratory study of a relatively new area; therefore, specific research questions or hypotheses were not defined before the study. It is possible that important user attitudes may have been omitted from the publicly available reviews. It is also likely that there may be an element of bias in publicly available user reviews, for example, iOS does not make all written reviews publicly available [57]. However, given the intricacy of reviews and the number of reviews analyzed, we are confident that our findings represent users’ likes and dislikes of mood-monitoring apps.

As app stores are very dynamic and frequently changing, the apps available, their features, and user review feedback are subject to change. Therefore, it is important for future research to develop effective methodologies that can rapidly evaluate user feedback within this field. We were able to automate the identification and extraction of apps and reviews; however, developing an automated analysis of user reviews would be a valuable advancement in future research.

### Conclusions

In this study, a content analysis framework was applied to a subsample of 1803 publicly available user reviews from 53 mood-monitoring apps appropriate for children and young people (based on app store age ratings). App store user reviews provide a valuable repository of anonymous, self-driven, and unstructured feedback. This paper provides a unique perspective on user attitudes and expectations toward mood-monitoring apps and allows an in-depth evaluation of the main features of engagement and potential barriers to adoption. Users value apps that can be personalized to their needs, have a simple and intuitive design, and allow accurate representation and review of complex and fluctuating moods.

Future studies should explore qualitative feedback from specifically recruited samples of children and adolescents using publicly available apps to explore whether the main features of engagement discovered in this study generalize to a defined child- and adolescent-only group and whether further details might be obtained from more reflective content. We hope these findings can support future guidelines on how apps are developed for end users, and we highlight the importance of including young people within the app design process to address disparities between end user perspectives and actual provisions within mHealth apps.

## Appendix

Multimedia Appendix 1 Description of application programming interface data collection methodology.

Multimedia Appendix 2 Full content analysis coding framework and illustrative quotes of all themes and subthemes identified.

Multimedia Appendix 3 List and meta data of all apps included in the analysis (n=53).

## Abbreviations

API: application programming interface
iOS: iPhone operating system
mHealth: mobile health
MRC: Medical Research Council
NIHR: National Institute for Health Research
TAM: technology acceptance model
