# Supplementary material for: User Perspectives of Mood-Monitoring Apps Available to Young People: Qualitative Content Analysis
Source: JMIR Mhealth Uhealth. 2020 Oct 10;8(10):e18140. doi: 10.2196/18140 (PMC7585773; doi:10.2196/18140)
Supplement: Multimedia Appendix 1 [file mhealth_v8i10e18140_app1.docx]

**Multimedia Appendix 1: Full Description of API Data Collection Methodology**

*Phase 1: Detecting mood-tracker apps using snowball sampling.*

Firstly we manually constructed a set of seed apps by using keyword searches ("mood tracker" and "mood monitor") on both Google Play and iOS. Seed apps were included which met the following inclusion criteria, i) self-reported mood monitoring or tracking was the apps primary purpose, ii) the apps were suitable for young people (under the age of 18) as described via the app store age rating, iii) the apps were not designed for a specific condition (other than mood disorders) (e.g. heart disease, high blood pressure, irritable bowel syndrome). 20 relevant apps were selected to use as seed apps.

We then used a snowball sampling method to collect mood-monitoring apps. This approach was built on the observation that most app stores suggest a series of similar apps on a given app's profile page. Such relational information has been shown to be useful in detecting functionally similar and competing apps on app stores [53, 54].

To eliminate apps that were irrelevant to mood trackers, we only included apps that contained "track mood", " monitor mood", "mood journal" or "mood diary" in their titles or descriptions during the snowball sampling procedures. We obtained 329 apps (221 apps from and 108 from App Store). For each app, we recorded all meta information returned by APIs, including an app's title, developer details, description, age rating, average star rating, number of user ratings, genre, URL and developer's details.

*Phase 2: Identifying relevant apps based on clustering analysis:*

Our second phase was to check the accuracy and refine the 329 apps. Following previous studies [36, 55], we identified relevant apps based on the content of each app's title. Unlike previous methods that rely on manual screening, we used a more efficient data-driven approach that combined clustering analysis and human annotations.

We first used an unsupervised clustering algorithm to group similar apps based on the content of their titles. Specifically, we characterized each app by a feature vector that contained terms in their titles and each term was weighted by widely used TFIDF [56]. Since the number of clusters were unknown in advance, we performed a hierarchical clustering algorithm [57] on these feature vectors to learn clusters of apps. The clustering algorithm generated 5 typical clusters of apps. Figure 1 shows the hierarchical clustering dendrogram of apps. We then inspected the most frequent terms in apps' titles of each cluster to understand these clusters. We found that the 5 clusters involved: (1) health management apps (N=51), (2) period trackers (N=51), (3) mental health self-treatment programs (N=93), (4) mood trackers (N=59), and (5) generic diary apps (N=75) respectively. By manually reviewing the full descriptions of all apps in relevant clusters, we found that most mood-monitoring apps were grouped within cluster 4. During this process, we identified 53 apps (accounting for 90% of all apps in cluster 4) that were highly relevant to mood-monitoring apps where 27 apps were from Google Play and 26 were from App Store.

Hierarchical clustering dendrogram of apps.


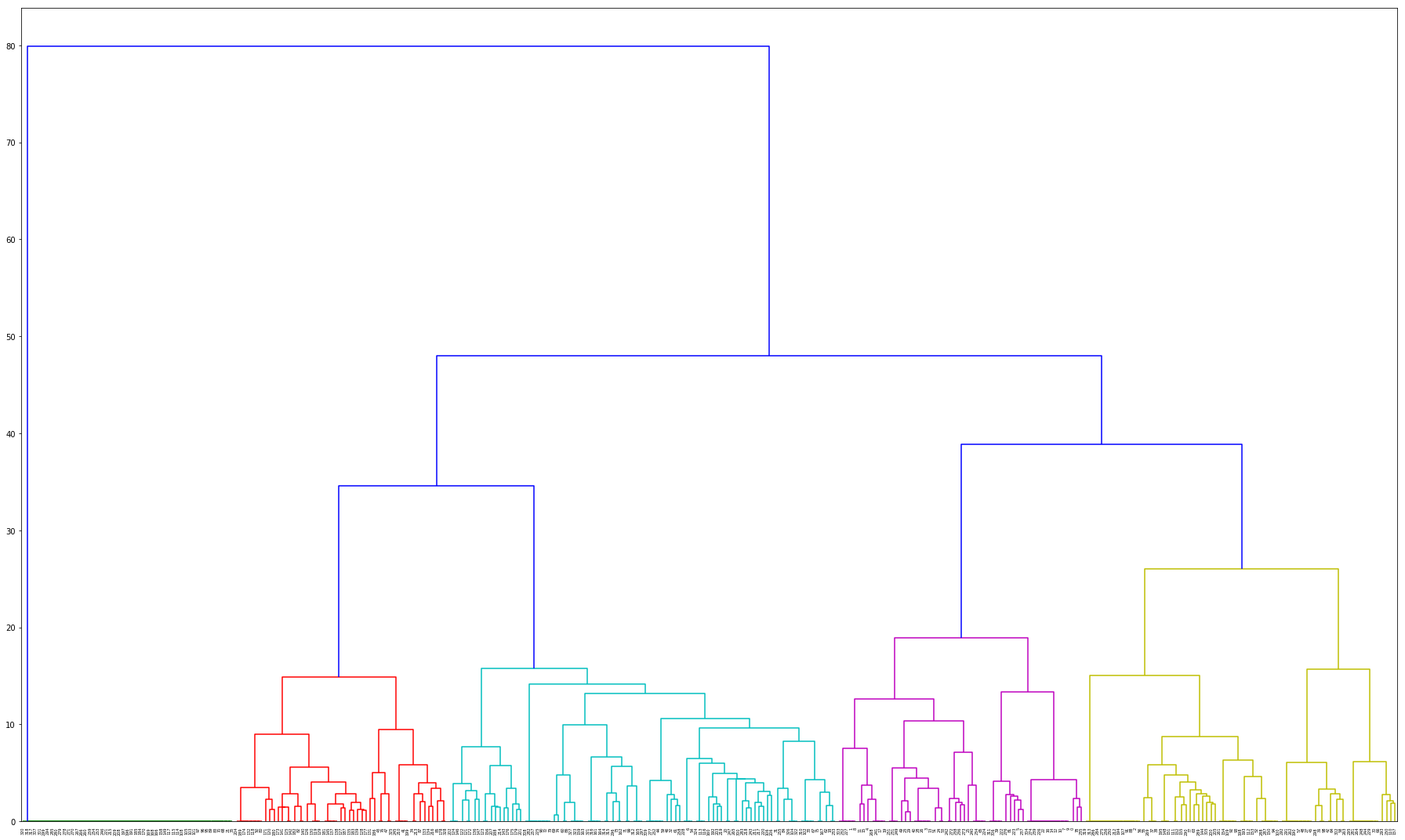


*Phase 3: Collecting user-generated reviews on apps*

Both platforms provide different ways in sorting reviews, such as by date (most recent), or by the helpfulness of a review rated by users (most helpful). This can lead to different sets of reviews for the same app on an app store. For a more comprehensive understanding of users' reviews, we collected the most recent and most helpful reviews, for all relevant apps on each app store. This also allows us to investigate the differences of reviews sorted in different ways. The numbers of reviews collected from Google Play and App Store by different sorting methods for our identified mood-monitoring apps are shown in Table 1.

Table 1 Numbers of reviews collected from each app store by different sorting methods.

| **Sorting method** | **Google Play** | **App Store** |
| --- | --- | --- |
| Most helpful | 13,593 | 2,232 |
| Most recent | 13,842 | 2,383 |

A Mann-Whitney test found no statistical difference between distribution of star ratings between reviews ordered by ‘most helpful’ or ordered by ‘most recent’ in terms of star ratings (Google Play Store: U= 279722039.5, *p* = 0.24; apple Store: Mann-Whitney U= 22411143.0 p=0.11). We therefore made the decision to focus this review on reviews rated as ‘most helpful’ due to the objective of finding features and functions that engage users most effectively.

We used scripts [38,39] to automatically collect reviews for the 53 mood-monitoring apps identified. While the APIs on Google Play allowed users to download all reviews for an app, iOS only allowed retrieval of up to 10 pages of reviews per country, where each page returned up to 50 reviews. To obtain a larger and more representative sample of reviews on the iOS store, we used a custom script to collect all 10 pages of reviews for each of the six largest English-speaking countries, namely US, UK, Canada, Australia, New Zealand, and Ireland.
