# Supplementary material for: User Perspectives of Mood-Monitoring Apps Available to Young People: Qualitative Content Analysis
Source: JMIR Mhealth Uhealth. 2020 Oct 10;8(10):e18140. doi: 10.2196/18140 (PMC7585773; doi:10.2196/18140)
Supplement: Multimedia Appendix 2 [file mhealth_v8i10e18140_app2.docx]

| Multimedia Appendix 2: Full content analysis coding framework and illustrative quotes of all themes and subthemes identified. | | | |
| --- | --- | --- | --- |
| Major theme | | **Quotes** | **Description/guidelines** |
| 1. Accessibility | |  | Any reference to the quality of app being easy to obtain or use including comments around convenience, simplicity and efficiency. Any mention of accessibility in terms of inclusiveness for people with disabilities (e.g. dyslexia).  **Exclude:** generic statements with no specific example of accessibility problems or opportunities for example ‘*Just plain does what it says it does’ (too generic).* |
| 1.1. Simple/easy to use | | “This app is simple and allows for easy thought record keeping”  “A clutter free interface” |  |
| 1.2. Efficiency | | “This app is an efficient and interesting way of keeping track of the day” |  |
| 1.3. Cost |  | “Finally a free app I would pay for!”  “I like the app but, when you have to pay it’s ridiculous!! I mean paying for mental and my own mental health?!’ |  |
| 1.4. Inclusivity | | “I have a coordination disorder so prefer a mobile app to a paper diary” |  |
| 2. Flexibility | | “The app gives you the ability to add emotions of your own, and has a much broader spectrum of emotion to choose from than other similar apps”  “It's precise and let's you rate as many times as you need to in one day and charts each individual mood”  “Would like to see some features like the ability to edit an entry rather than delete it and resubmit”.  ‘I use it to vent...I haven’t reached a character limit yet...and I am grateful for that!’. | Any reference to flexibility or options within the app. Flexibility includes user comments on the ability to customize or personalize a certain aspect of the app, for example, custom emotions or , choosing a color theme. |
| 3. Technical features |  |  | Any mention of a technical app feature either structural or functional including comments on data, bugs and security features.  **Exclude:** generic technical comments that do not specify a particular feature of engagement or technical issue for example *‘loved it a lot before the newest update, still like it just not as much’* (no specific technical feature mentioned) |
| 3.1. Data loss/backup/saving | | “I loved this app! UNTIL I discovered that after using it for a few months it was deleting all of my entries each month. When I figured this out I was super upset, it was like someone deleted my journal. This sucks.” |  |
| 3.2. Bugs/display errors/  app entry issues | | “Hi, i don't see the day. I was about to input my emotion when I don't see the dates.” |  |
| 3.3. Export/share/sync | | “Wish it would export mood entries as well the mood graph I thought that this mood tracker fit most of my needs for information that I would like to track but when I went to export the data to share with my healthcare provider, I saw that it exports the graph but none of the details! I think that it would be a much more powerful tool if we could share that information as well.” |  |
| 4. Recording and representation of mood | | “I have multiple diagnoses so it has been very helpful to be able to create custom scales. The way these scales work, with the user rating a number of different factors using sliders instead of discrete numbers, makes it easy to derive clear results from a complex array of both physical and emotional indicators”  “I like the app but am dissatisfied with the number of choices for the moods, it would be nice to have an "other" option where we can put a mood name of our choice.”  “Vague enough that they don't box you in, but specific enough to still be useful.”  “The emotions listed are extremely helpful and when I’m confused about my own emotions, the layout helps me pick.” | Any comment relating to how users record or represent their mood within the app. This can include answering a questionnaire selecting an emotion or emoji, writing notes, adding photos or videos. |
| 5. User requests | | “My only request would be to have at least a basic social aspect.”  “My one concern is not being able to edit or delete tags once you create them. Is there a way to do this?” | Any user request for an additional feature or function or a request to the developer to fix an error/bug.  **Exclude:** user responses to the developer thanking them for responding to a request (code as developer). |
| 6. Reflecting on mood | | “Big fan of this app. Helps me track my ups and downs, to make more informed decisions about what to change on a day-to-day basis.”  “I have a tendency to forget when I have had good moments when I'm in a bad mood and vice versa. This has really helped to keep an eye on and better monitor my mental health.”  “The graphs are great for identifying a few bad days vs longer trends” | Any comment relating to the user reflecting on their mood when using the app. For example, identifying patterns in mood, mood graphs, behavioral change, reflecting on good/bad days.  **Exclude:** generic comments just restating the purpose of the app, for example “*great for mood monitoring*’ or ‘*great for logging your mood each day*”. |
| 7. Design | | “Sleek UI. Pretty cool design. And a beautiful concept.”  ”Kudos to the developers for their work and their design, I like the minimal and easy to navigate UI.”  “Its extremely simple but i think that’s is why i like it. More features may make the app cluttered.” | Any reference to the design of the app with specific reference to the user interface, display screen or app layout. Include references to app aesthetics.  **Exclude**: generic design statements that do not mention a specific feature for example, “*really unique and well-made app.”* |
| 8. Health promotion | |  | Any mention of the app acting within a therapeutic or cathartic capacity, for example relieving tension, feeling calm and dealing with emotions.  Also comments of the app helping users in their ongoing therapy (e.g. counselling sessions) |
| 8.1. Therapeutic | | “It helps me work on self-care and seeing what i need to work on mentally and be a healthy and happy soul a god send’, ‘It really helps me concentrate on good things that happened and take my mind off bad stuff.” |  |
| 8.2. Health professional | | “The simple words that I use to describe myself in this app best meet my needs and allow me to more clearly convey my life to my doctor and counsellor”.  “I’m able to email my results to my therapist, and it helps us to focus on specific areas of concern.” |  |
| 8.3. Psychoeducation | | “I like the teaching component to help me interpret and understand cog. B. T.” |  |
| 8.4. Signposting | | “I appreciate the resource tab a lot during times of distress.” |  |
| Minor Themes |  | **Quotes** | **Description/Guidelines** |
| 1. Notifications and reminders | | “Its daily reminders really help you focus on the good things that happen and I find, even on bad days it encourages me to find something positive.” | Any positive or negative reference to app notifications or reminders, for example frequency of reminders, helpfulness of reminders, personalisation of reminders. |
|  |  | “Love that I can set my reminders to exactly when I want them to pop up...keeps me on track.” |  |
| 2. Recommendation | | “Would recommend to others/Therapist told me to.” | Any recommendation of the app made by the user or users describing being recommended the app (e.g. by therapist). |
| 3. Privacy, security, and transparency | | “Removed it quicker than I could install it. it seems the only way to create an account is to have Facebook. I do not use Facebook because of privacy concerns.” | Any reference to the transparency or openness of app content, for example, clear and transparent app information or subscription and payment transparency. Reference to privacy and security features on the phone. |
|  |  | “Only way to create an account is if it’s connected to Facebook. With such personal information being stored on the app, I’m not sure I trust it to not sell that off as profit.” |  |
|  |  | “I honestly like this app. But I am a bit worried about the security of my data. App description does not mention anything about whether user data is stored on the cloud in encrypted form or not.” |  |
|  |  | “Zero mentions of encryption. Several mentions of how your data could end up on a server in different countries. I am honestly disgusted by this. They want you to subscribe to the app and then are going to use your very private data to make even more money off of you.” |  |
| 4. Developer |  | “I still haven’t gotten a response from the developer about why my entries got deleted.”  “I've been impressed with the developer's dedication to maintaining and improving this app.”  “I’ve emailed the developers with no reply so my only course of action is to leave a negative review.” | Any reference to developer responsiveness, maintenance/improvement of the app, and the developer’s response to specific user requests. |
| 5. Adverts |  | “I really appreciate that you can choose if you want to watch an ad and support the developer”  “The ads when submitting a report are disruptive, and block where you're typing.”  “There are ZERO ads. It works perfectly. I legitimately press the option to watch adds because this app is gorgeous with absolutely zero ads.” | Any mention of adverts, for example, amount, ability to watch and advert options. |
| 6. Social or community |  | “It becomes a virtual network to support yourself and others”.  “If only you could type a response, not only emojis because I usually want to say something encouraging but I never can.” | Any reference to a peer or community support aspect to the app, for example, chat bot within the app for interaction/online social forums. |
