# Supplementary material for: User Perspectives of Mood-Monitoring Apps Available to Young People: Qualitative Content Analysis
Source: JMIR Mhealth Uhealth. 2020 Oct 10;8(10):e18140. doi: 10.2196/18140 (PMC7585773; doi:10.2196/18140)
Supplement: Multimedia Appendix 3 [file mhealth_v8i10e18140_app3.docx]

| Multimedia Appendix 3: List and meta data of all apps included in the analysis (n=53) | | | | | | |
| --- | --- | --- | --- | --- | --- | --- |
| App Title | No.  Reviews | Content Age Rating | Genre | No. Star  Ratings | Average Star Rating | Price |
| iOS APPS |  |  |  |  |  |  |
| Activity and Mood Diary by Ginsberg | 37 | 4+ | Health & Fitness | 12 | 1.5 | Free |
| Breeze: mood journal, diary | 54 | 4+ | Health & Fitness | 212 | 4.5 | Free |
| Emojion Journal & Mood Diary | 22 | 4+ | Lifestyle | 28 | 4.5 | Free |
| Emoly - Personal mood tracker | 3 | 4+ | Lifestyle | 0 | 5 | $0.99 |
| eMoods Bipolar Mood Tracker | 68 | 4+ | Lifestyle | 1411 | 5 | Free |
| Feelic - Mood Tracker, Share,Text & Chat with Friends | 6 | 4+ | Lifestyle | 7 | 4 | Free |
| Feelings Diary - Mood Journal | 37 | 4+ | Lifestyle | 345 | 4.5 | Free |
| iMoodJournal - Mood Diary | 831 | 4+ | Lifestyle | 104 | 4.5 | $2.99 |
| Mood - Journal & Anxiety Chat | 198 | 12+ | Health & Fitness | 1398 | 4.5 | Free |
| Mood D - Your emotional diary | 18 | 4+ | Lifestyle | 5 | 4.5 | Free |
| Mood Log - Track your Mood | 3 | 4+ | Medical | 0 |  | Free |
| Mood Pixel: Year in Pixels | 23 | 4+ | Lifestyle | 34 | 5 | Free |
| Mood Ring: Your Emoji Journal | 15 | 4+ | Health & Fitness | 42 | 4.5 | Free |
| Mood: Track your mood | 3 | 4+ | Health & Fitness | 38 | 4.5 | Free |
| Moodfit-Shape Up Your Mood | 85 | 12+ | Health & Fitness | 90 | 5 | Free |
| Moodistory Mood Tracker, Diary | 11 | 4+ | Health & Fitness | 25 | 5 | $3.99 |
| MoodKit - Mood Improvement Tools | 441 | 12+ | Health & Fitness | 125 | 4.5 | $4.99 |
| Moodly: Mood Tracker & Journal | 3 | 4+ | Lifestyle | 7 | 4.5 | Free |
| MoodWell - daily mood journal | 9 | 4+ | Lifestyle | 30 | 5 | Free |
| Moody - Daily Mood Tracker | 93 | 4+ | Lifestyle | 151 | 4 | $1.99 |
| Moody: Mood Tracker & Journal | 14 | 4+ | Lifestyle | 69 | 4.5 | Free |
| My Life My Voice Mood Journal | 41 | 4+ | Lifestyle | 22 | 4.5 | Free |
| Scribio - Elegant Mood Journal | 2 | 4+ | Lifestyle | 5 | 4.5 | Free |
| T2 Mood Tracker | 146 | 4+ | Health & Fitness | 15 | 4 | Free |
| Tracker-Mood & Energy Diary | 7 | 4+ | Health & Fitness | 30 | 4.5 | $1.99 |
| Wellness: Mood Meds & Health | 49 | 12+ | Health & Fitness | 219 | 4.5 | Free |
| Google Play Apps |  |  |  |  |  |  |
| aiMei - Personality Tests & Mood Tracking | 290 | Everyone | Lifestyle | 1365 | 4.5 | Free |
| Chat Journal - Timeline Diary/Journal Mood Tracker | 96 | Everyone | Lifestyle | 183 | 4.7 | Free |
| Daylio - Diary, Journal, Mood Tracker | 4379 | Everyone | Lifestyle | 243514 | 4.8 | Free |
| Diaro - Diary, Journal, Notes, Mood Tracker | 4379 | Everyone | Productivity | 80231 | 4.5 | Free |
| Diary - Mood Tracker | 20 | Everyone | Lifestyle | 48 | 3.7 | Free |
| Diary, mood tracker | 37 | Everyone | Lifestyle | 183 | 4.2 | Free |
| eMoods Bipolar Mood Tracker | 1485 | Everyone | Health & Fitness | 3863 | 4.4 | Free |
| Free Mood Tracker | 36 | Everyone | Health & Fitness | 105 | 4 | Free |
| Gratitude Journal: Habit & Mood Tracker- hiMoment | 96 | Everyone | Health & Fitness | 665 | 4.1 | Free |
| iDaily Mood - Simple, Easy and Clean Mood Tracker | 8 | Everyone | Lifestyle | 10 | 4.2 | Free |
| Jade - Mood Tracker, Diary, Journal | 65 | Everyone | Lifestyle | 126 | 4.7 | Free |
| Journee - Diary, Journal, Mood Tracker, Notes | 379 | Everyone | Lifestyle | 952 | 4.5 | Free |
| Memorize - Diary, Journal, Mood Tracker | 285 | Everyone | Lifestyle | 549 | 4.8 | Free |
| Mood diary - Track emotions | 50 | Everyone | Lifestyle | 80 | 4.2 | Free |
| Mood Log | 486 | Everyone | Health & Fitness | 1082 | 4.4 | Free |
| Mood Pixel: Year in Pixels Diary & Mood Tracker | 54 | Everyone | Health & Fitness | 103 | 4.7 | Free |
| Mood Tracker | 21 | Everyone | Lifestyle | 31 | 3.6 | Free |
| Mood Tracker | 35 | Everyone | Medical | 110 | 3.8 | Free |
| Mood Tracker - Bipolar Mood Journal, Mood Diary | 5 | Everyone | Lifestyle | 11 | 3.5 | Free |
| Mood Tracker & Questions Diary (anti depression) | 25 | Everyone | Lifestyle | 671 | 4.7 | Free |
| Mood-Diary | 3 | Everyone | Health & Fitness | 16 | 3 | Free |
| MoodiModo Mood Tracker | 46 | Everyone | Health & Fitness | 161 | 4 | Free |
| My Diary - Daily Notes, Journal & Mood Tracker | 259 | Everyone | Productivity | 487 | 4.6 | Free |
| PAX Mood Tracker | 6 | Everyone | Health & Fitness | 11 | 4.4 | Free |
| Puncher - Diary, Mood Tracker, Diet Journal | 80 | Everyone | Lifestyle | 2766 | 4.5 | Free |
| Stories - Timeline Diary / Journal, Mood Tracker | 178 | Everyone | Lifestyle | 526 | 4.4 | Free |
| T2 Mood Tracker | 733 | Everyone | Health & Fitness | 1803 | 4 | Free |
